# A Functional Interface at the rDNA Connects rRNA Synthesis, Pre-rRNA Processing and Nucleolar Surveillance in Budding Yeast

**DOI:** 10.1371/journal.pone.0024962

**Published:** 2011-09-19

**Authors:** Nathalie Leporé, Denis L. J. Lafontaine

**Affiliations:** 1 RNA Metabolism, Fonds de la Recherche Scientifique (FRS-FNRS), Université Libre de Bruxelles, Charleroi-Gosselies, Belgium; 2 Center for Microscopy and Molecular Imaging (CMMI), Académie Wallonie–Bruxelles, Charleroi-Gosselies, Belgium; Victor Chang Cardiac Research Institute (VCCRI), Australia

## Abstract

Ribogenesis is a multistep error-prone process that is actively monitored by quality control mechanisms. How ribosomal RNA synthesis, pre-rRNA processing and nucleolar surveillance are integrated is unclear. Nor is it understood how defective ribosomes are recognized. We report in budding yeast that, *in vivo*, the interaction between the transcription elongation factor Spt5 and Rpa190, the largest subunit of RNA polymerase (Pol) I, requires the Spt5 C-terminal region (CTR), a conserved and highly repetitive domain that is reminiscent of the RNA Pol II C-terminal domain (CTD). We show that this sequence is also required for the interaction between Spt5 and Nrd1, an RNA specific binding protein, and an exosome cofactor. Both the Spt4-Spt5, and the Nrd1-Nab3 complexes interact functionally with Rrp6, and colocalize at the rDNA. Mutations in the RNA binding domain of Nrd1, but not in its RNA Pol II CTD-interacting domain, and mutations in the RRM of Nab3 led to the accumulation of normal and aberrant polyadenylated pre-rRNAs. Altogether these results indicate that Nrd1-Nab3 contributes to recruiting the nucleolar surveillance to elongating polymerases to survey nascent rRNA transcripts.

## Introduction

Ribosome synthesis is a complex pathway that involves hundreds of individual error-prone reactions (reviewed in [1]). To circumvent problems associated with the accumulation of aberrant precursor ribosomes and defective mature particles, cells have evolved multiple, complementary, quality control mechanisms (reviewed in [2]). Nucleolar surveillance ensures the rapid and specific recognition of unfaithful pre-ribosomes, e.g. those carrying improperly processed, misfolded, or hypomodified pre-rRNAs, and their targeting for decay. Nucleolar surveillance is evolutionarily conserved and involves both 5′-3′ ([3], [4]) and 3′-5′ RNA decay pathways ([5], [6], [7]). The biomedical relevance of rapidly clearing the cells of damaged, and potentially harmful, ribosomes is exemplified by the observation that chemically-altered ribosomes accumulate in the neurons of patients suffering from Alzeihmer's disease (reviewed in [2]). Western honeybees, a major crop pollinator, suffering from colony collapse disorder (CCD) accumulate polyadenylated rRNAs ([8]), a hallmark of ribosome degradation, which also highlights the importance to understand ribosome surveillance mechanisms.

Pre-ribosomes are largely assembled co-transcriptionally, resulting in the illustrious ‘Miller spreads’ Christmas tree images (for examples see [9]). Components of the RNA synthesis and processing machineries are, therefore, spatially and temporally closely related and poised to interact functionally. The best demonstration that rRNA synthesis and pre-rRNA processing are functionally connected processes is provided by the observation that mutations affecting RNA polymerase (Pol) I elongation also impact pre-rRNA cleavage ([10], [11], [12]). The Spt4-Spt5 complex, which was originally characterized as an RNA Pol II transcription elongation factor ([13]), later turned out to physically interact also with RNA Pol I ([10], [14], [15]), and to impact RNA Pol I function ([10], [12]). The Spt4-Spt5 complex influences RNA Pol I transcription both positively and negatively, and alters pre-rRNA processing kinetics ([10], [12]). The kinase Grc3 provides additional evidence for connections between pre-rRNA synthesis and cleavage, since it is required for both RNA Pol I termination by a mechanism known as ‘torpedo’ and for pre-rRNA processing ([16], [17]).

A recurrent theme in the field of RNA research is that *trans*-acting RNA processing factors are often shared between pathways involving distinct classes of RNAs; this has been best illustrated for exoRNases (discussed in [18]). It ensues that there is a certain level of commonalities between otherwise distinctive RNA pathways. A nucleolar 3′-5′ rRNA surveillance pathway was recently described in which aberrant precursor rRNAs are selected for degradation by the RNA exosome following the addition of short poly(A) tails by TRAMP complexes ([5], [6]). Inhibition of early pre-rRNA processing reactions in yeast, and alteration in RNA Pol I function in mouse, generate aberrant rRNA precursors that are substrates of this surveillance ([5], [6], [7]). The heterotrimeric Nrd1-Nab3-Sen1 complex, which has been involved in the termination of transcription of short noncoding RNA Pol II transcripts, such as snRNAs, snoRNAs, and CUTs (reviewed in [19], [20]), interacts with both Spt5 and the exosome ([21]). In transcription termination, Nrd1-Nab3-Sen1 is recruited to nascent RNA Pol II transcripts through the interaction of Nrd1 with the phosphorylated CTD of RNA Pol II, and through the direct and cooperative binding of both Nrd1 and Nab3 to short ubiquitous RNA motifs, also abundantly present in large RNAs, such as ribosomal RNAs. The recent involvement of Nrd1-Nab3 in the metabolism of RNA Pol III transcripts support the view that this complex carries functions independent of RNA Pol II termination ([22]).

In this work, we were interested in testing whether the sequence specific RNA-binding and exosome-interacting Nrd1-Nab3-Sen1 complex might contribute to the recruitment of nucleolar surveillance to deficient ribosomal transcripts, independently of its function in the termination of short RNA Pol II transcripts, and in better understanding how the Spt4-Spt5 complex, which interacts with both RNA Pol I and Nrd1, connects pre-rRNA synthesis, pre-rRNA processing and ribosome surveillance.

## Results

### Ribosomal RNA precursors are stabilized in *nrd1* and *nab3* mutants

Previous work in budding yeast and mammals established that aberrant rRNA fragments resulting from abortive RNA synthesis or unfaithful pre-RNA processing, are targeted for degradation by a nucleolar surveillance pathway involving the TRAMP and exosome complexes ([5], [6], [7]). We were interested in finding out whether additional nuclear exosome cofactors contribute to this pathway. Potential candidate proteins included the Nrd1-Nab3-Sen1 complex. Putative binding sites for both Nrd1 and Nab3 are present on pre-rRNAs (Fig S1), and Nrd1 interacts with the RNA Pol I elongation factor Spt5 and the nuclear exosome ([21]). The interaction between the exosome and Spt5 is evolutionarily conserved ([23]).

**Figure 1:**
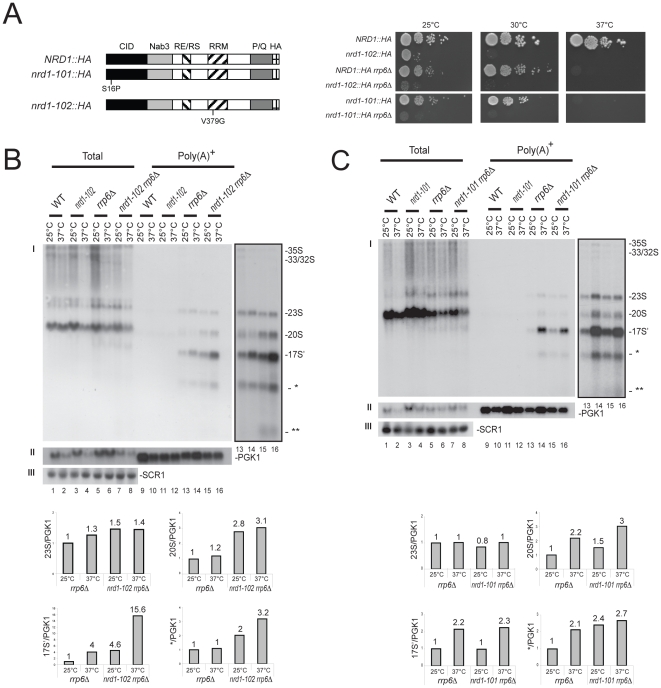
Involvement of the RRM and CID domains of Nrd1 in nucleolar surveillance. **A**, (left) Domain mapping of Nrd1. All three *nrd1* alleles used in this work (provided by Prof. Jeff Corden, Johns Hopkins University School of Medicine) are HA-epitope tagged at the carboxyl terminal end of the protein, which allowed us to establish that neither mutation affects protein stability (see Fig S2B and C). Nrd1 consists of an amino terminal RNA Pol II CTD interacting domain (CID), followed by a Nab3-interacting domain (Nab3), a central RE/RS-rich domain (RE/RS), an RNA binding domain (RRM), and a carboxyl terminal P/Q-rich-domain. The amino acid substitutions responsible for the thermosensitive phenotypes for growth in the conditional alleles, *nrd1-101* and *nrd1-102,* are indicated. (right) Ten-fold serial dilutions of yeast cultures. Cells were grown on solid rich medium at the indicated temperatures for 2 d (30°C and 37°C) or 3 d (25°C). The Nrd1-HA-tagged wild-type control construct was fully functional. **B,** Analysis of the RRM mutation. (top) Northern-blot analysis of total and purified poly(A)^+^ RNAs in different mutant background at the indicated temperatures. Poly(A)^+^ RNA was purified on poly-dT-coated magnetic beads (see [6]). Total RNA and poly(A)^+^ RNAs were loaded in a 1∶50 ratio, separated on 1.2% agarose/6% formaldehyde gel, transferred to nylon membranes and hybridized. Cells were grown to mid-log phase at 25°C and then transferred to the non-permissive temperature for 2 h. The probe used to detect the pre-rRNAs was LD471 (panel I, see Fig S3A). Total RNA was normalized to SCR1. Poly(A)^+^ RNA was normalized to the *PGK1* mRNA. (bottom) Phosphor Imager quantitation. Histograms represent the relative distribution of specific RNA normalized to the *PGK1* mRNA in different mutant background at different temperatures. **C,** Analysis of the CID mutation. Legend as in panel B. See also Fig S2.

Thermosensitive mutations have been described that affect Nrd1 either in its RNA Pol II CTD-interacting domain (CID, *nrd1-101*) or in its RNA recognition motif (RRM, *nrd1-102*) (Fig 1A, [24], [25]). In particular, the *nrd1-102* allele carries a single point mutation in the RRM leading to a deficiency in snoRNA termination as a result of reduced RNA binding activity ([24]). We noted that the temperature-dependent growth defects observed in cells carrying the *nrd1* mutations were strongly exacerbated in the absence of Rrp6, indicating genetic interactions (Fig 1A).

An aberrant polyadenylated rRNA precursor, 17S', extending from within the 18S rRNA coding sequence to site A_3_ in the internal transcribed spacer 1 (ITS1), is known to strongly accumulate in strains inactivated for *RRP6* ([5], [6], [26]). Initially, we narrowed the 5′-end of this precursor by serial Northern-blot hybridizations on purified polyadenylated RNAs to a position lying between +1100 and +1150, with respect to the transcription start site (data not shown). Throughout this work, we have then used the steady-state accumulation of polyadenylated 17S' RNA as an indicator of nucleolar surveillance activity. In particular, we have analyzed whether the amount of polyadenylated 17S' detected in the absence of Rrp6 is altered upon Nrd1, inactivation, and whether specific functional domains of Nrd1 are involved (Fig 1B and C). In these analyses, the amount of total RNA loaded on the gels was standardized to SCR1 (panels III, lanes 1–8 in both B and C), and the amount of purified polyadenylated RNA was standardized to the PGK1 mRNA (panels II, lanes 9–16 in B and C). *GRE1* readthrough transcripts were stabilized in *nrd1-101* and in *nrd1-102* mutants, as expected, and we notably found that the concomitant inactivation of Rrp6 and Nrd1 had synergistic effects on short-lived transcripts stabilization (Fig S2).

The inactivation of Rrp6 led to the accumulation of polyadenylated pre-rRNA precursors, as previously described (Fig 1, panels B and C, lanes 13 and 14, [6], [26]). These included physiological RNA substrates that normally enter the pre-rRNA processing pathway (such as the 20S pre-rRNAs, see Fig S3), the aberrant precursor 23S, that results from premature cleavage in ITS1, and truncated rRNA fragments, originating from unfaithful pre-rRNA processing (17S', * and **).

The inactivation of the RRM domain of Nrd1 led to the striking stabilization of the 17S' and shorter rRNA fragments, labeled * and ** (Fig 1B, see inset and quantitation). The 20S pre-rRNA, the immediate precursor to the 18S rRNA, was also stabilized, indicating, quite surprisingly, that under physiological conditions a significant fraction of this precursor is degraded. The most striking stabilization was for the 17S' RNA, which was >3-fold. The inactivation of the CID domain of Nrd1 (Fig 1C) did not lead to such a striking stabilization of the 17S' RNA, indicating importantly that the function of Nrd1 in rRNA precursor stabilization is independent from that involving its binding to the RNA polymerase II CTD (see Discussion). Note that in the *nrd1-101* and *nrd1-102* alleles, the thermo-inactivation of Nrd1 did not affect protein stability, as established by Western-blot analysis (Fig S2B and C).

**Figure 2:**
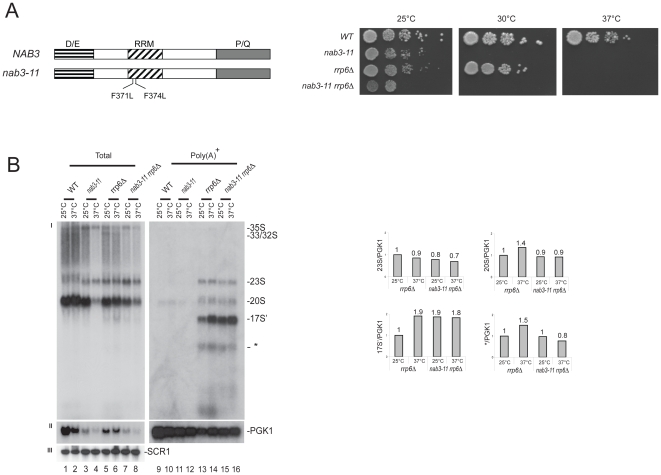
Involvement of the RRM domain of Nab3 in nucleolar surveillance. **A,** (left) Domain mapping of Nab3. Nab3 consists of an amino terminal D/E-rich, a central RRM and a carboxyl terminal P/Q-rich domain. The two amino acid substitutions in the RRM domain responsible for the thermosensitive phenotypes in the *nab3-11* are indicated (provided by Prof. Jeff Corden, Johns Hopkins University School of Medicine). (right) Ten-fold serial dilutions of yeast cultures. Cells were grown on solid rich medium at the indicated temperatures for 2 d (30°C and 37°C) or 3 d (25°C). **B,** Analysis of the Nab3 RRM mutation. (left) Northern-blot analysis of total and purified poly(A)^+^ RNAs in different mutant background at the indicated temperatures (see legend to Fig 1). (right) Phosphor Imager quantitation. Histograms represent the relative distribution of specific RNA normalized to the *PGK1* mRNA in different mutant background at different temperatures.

To test further the involvement of the Nrd1 complex in the targeting for decay of polyadenylated rRNAs, we examined the effects of alterations in an important partner: Nab3 (Fig 2). Analysis with the thermosensitive allele *nab3-11*, carrying two point mutations within the RRM region (Fig 2A and [24]), indicated that Nab3 and Rrp6 interact genetically and biochemically. Upon concomitant inactivation of Rrp6 and the RRM of Nab3, the growth inhibition was exacerbated at 25°C, and the 17S' RNA was further stabilized at 25°C (Fig 2A and Fig 2B, compare lanes 13 and 15). *GRE1* readthrough transcripts were stabilized in *nab3-11* mutants, as expected, and we observed that the stabilization was stronger when *RRP6* was also inactivated (Fig S2E).

The *nrd1* and *nab3* mutations only affected marginally, and to the same extent, the steady-state accumulation of mature rRNAs (data not shown). However, interestingly, only the *nab3-11* mutation impacted strongly pre-rRNA processing, not the *nrd1-101* or *nrd1-102* mutations (compare lanes 3 and 4 in panel I in Figs 1B and 1C with Fig 2B, and data not shown). This indicates that Nab3 carry additional functions that can either take place within, or independently from, the Nrd1-Nab3 complexes.

### The Nrd1-Nab3 and Spt4-Spt5 complexes colocalize at the rDNA locus

**Figure 3:**
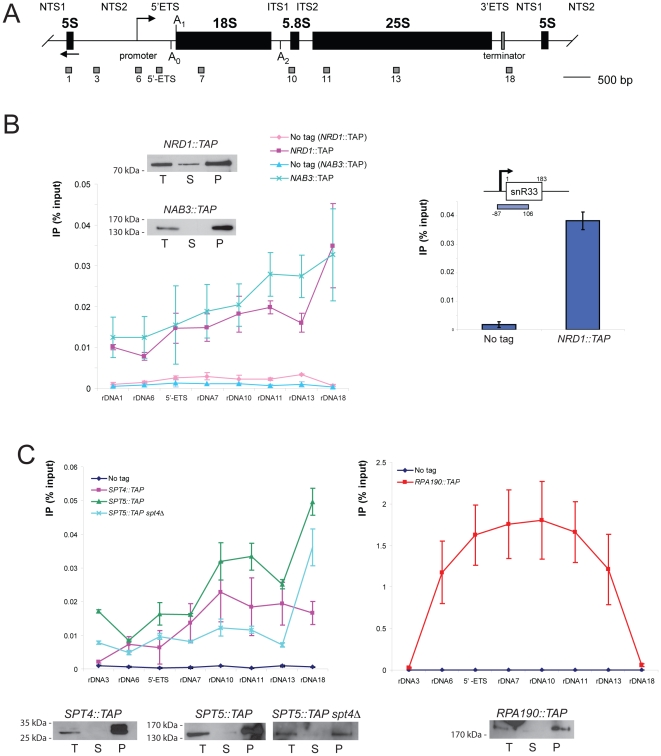
The Nrd1-Nab3 and Spt4-Spt5 complexes colocalize at the rDNA with a particular enrichment towards the 3′-end of the gene. A, Yeast rDNA unit and amplicons used in the ChIP analysis. The RNA Pol I transcript extends from the promoter to the terminator regions of the rDNA and encodes the 18S, 5.8S, and 25S rRNAs. Mature rRNAs are interspersed with external (5′- and 3′-ETS) and internal (ITS1 and ITS2) transcribed spacers. The 5S gene is transcribed by RNA Pol III in the opposite direction and is embedded in nontranscribed regions 1 and 2 (NTS1 and NTS2). Amplicons used in the ChIP analysis are indicated (1, 3, 6, 5′-ETS, 7, 10, 11, 13, 17, and 18). Scale bar is 500 bp. B, The Nrd1-Nab3 complex interacts with the rDNA. Chromatin extracts prepared from yeast cells expressing a functional carboxyl terminal TAP-tagged version of Nrd1 (*NRD1::TAP*) or Nab3 (*NAB3::TAP*), and an otherwise isogenic wild-type strain (No tag), grown to mid-log phase at 30°C, were submitted to coprecipitation analysis with IgG-coated magnetic beads, and the copurifying DNA were analyzed by qPCR with oligonucleotide pairs specific to the entire stretch of the rDNA locus (see panel A). Enrichment at each position is indicated (n = 3). Importantly, chromatin was extracted from independent cultures and used in independent coprecipitation analysis (biological triplicate). Inset: Western-blot analysis of protein coprecipitation efficiency. Chromatin extracts from the total (T), supernatant (S), and pellet (P) fractions were loaded in a 1∶1∶10 ratio. Membranes were probed with an anti-ProtA antibody. Right panel: the interaction of Nrd1 at the snR33 locus was established as a control. The cartoon depicts the *snRN33* locus with its promoter (arrow) and the amplicon (−87 to 106) used in the qPCR. For the left panel, p-values were calculated with a standard paired t-test, and are as follows: *NRD1::TAP*: 0.0039 (rDNA1), 0.012 (rDNA6), 0.0347 (5′-ETS), 0.0357 (rDNA7), 0.0348 (rDNA10), 0.0032 (rDNA11), 0.0119 (rDNA13), 0.0297 (rDNA18); for *NAB3::TAP*: 0.0475 (rDNA1), 0.0495 (rDNA6), 0.1256 (5′-ETS), 0.0408 (rDNA7), 0.0229 (rDNA10), 0.0109 (rDNA11), 0.0102 (rDNA13), 0.0372 (rDNA18). C, The Spt4-Spt5 RNA polymerase elongation complex interacts with the rDNA and the interaction of Spt5 with the rDNA is reduced in the absence of Spt4 (left). See legend to panel B for details. As a control, the interaction of a functional carboxyl terminal TAP-tagged version of the largest subunit of RNA Pol I (Rpa190) at the rDNA was established (right). n = 3, except for Rpa190 and Spt4 (n = 2). Lower panels: Western-blot analysis of protein coprecipitation efficiency. p-values for *SPT5::TAP* are 0.0231 (rDNA3), 0.0436 (rDNA6), 0.0258 (5′-ETS), 0.0163 (rDNA7), 0.0252 (rDNA10), 0.0134 (rDNA11), 0.0253 (rDNA13), 0.0239 (rDNA18); for *SPT5::TAP spt4Δ*: 0.013 (rDNA3), 0.0317 (rDNA6), 0.0187 (5′-ETS), 0.0068 (rDNA7), 0.0146 (rDNA10), 0.0074 (rDNA11), 0.01 (rDNA13), 0.0171 (rDNA18).

Nrd1 and Nab3 have previously been shown to stabilize RNA Pol II transcripts originating from the rDNA intergenic sequence (IGS) and UV cross-linking has recently established that Nrd1 and Nab3 directly interact with these short-lived RNAs, as well as with RNA Pol I and RNA Pol III transcripts ([22], [27]). Altogether this indicates that Nrd1 and Nab3 are physically present at the rDNA. To expand upon these analyses, we performed a high-resolution chromatin immunoprecipitation (ChIP) analysis across the rDNA locus in cells expressing functional carboxyl terminal TAP-tagged constructs of Nrd1 or Nab3 (Fig 3B). Both Nrd1 and Nab3 were efficiently crosslinked across the rDNA unit (as compared to untagged control strains), with particular enrichment towards the 3′-end of the rDNA gene (terminator, amplicon 18); this is where the IGS transcripts are synthesized by RNA Pol II in the opposite direction to the RNA Pol I gene. Both Nrd1 and Nab3 were also efficiently colocalized at the 5′-end of the 25S gene (amplicon 11), where the SSU-processome comes in close proximity to the rDNA following nascent pre-ribosome compaction ([6], [9]). The SSU-processome is a snoRNP-based macromolecular complex involved in the early nucleolar cleavages of the pre-rRNAs at sites A_0_–A_2_ (reviewed in [2]). The efficiency of coprecipitation was tested by Western-blotting; both Nrd1 and Nab3 were efficiently recovered in the chromatin pellets (see inset panel B). As a control for ChIP specificity, Nrd1 was colocalized at the snR33 locus, where it is required for snoRNA termination (see [28], [29]).

Previously, the research teams of Profs Nomura and Schneider established commonalities in the process of transcription elongation by RNA Pol I and RNA Pol II ([10], [12], [15], [30], [31]). In particular, the Spt4-Spt5 heterodimer (known as the *D*RB *s*ensitivity-*i*nducing *f*actor, or DSIF, in humans), which interacts physically and genetically with RNA Pol II and is required for RNA Pol II elongation ([13], [32], see Discussion), was shown to also interact with core RNA Pol I subunits both *in vitro* (Rpa34.5, Rpa49, Rpa135 and Rpa190) and *in vivo* (Rpa135 and Rpa190), and to colocalize to the rDNA ([10], [15]). Interaction between Spt5 and three subunits of RNA polymerase I (Rpa190, Rpa135 and Rpa49) had also been reported *in vivo* by the team of Prof. Harzog ([14]). In addition, Spt4 and Spt5 were recently shown to interact functionally with Rpa49 ([12], [15]).

We confirmed the colocalization of the Spt4-Spt5 complex at the rDNA by ChIP analysis in strains expressing functional, epitope-tagged, versions of Spt4 or Spt5 (Fig 3C). Both proteins were enriched across the rDNA with peaks starting at the middle towards the 3′-end of the gene, and showed similar interaction profiles. Spt4 is encoded by a nonessential gene, which allowed us to test its requirement for the interaction of Spt5 with the rDNA. In the absence of Spt4, the interaction of Spt5 at the rDNA was reduced (Fig 3C). As described above, ChIP efficiency was established by Western blotting on chromatin fractions. As a further control, a core subunit of RNA polymerase I (Rpa190) was coprecipitated and shown to colocalize precisely with the coding sequence, covered by amplicons 6 to 13 (Fig 3C).

### The CTR domain of Spt5 is required for the interactions between Spt5 and Nrd1 and between Spt5 and Rpa190

**Figure 4:**
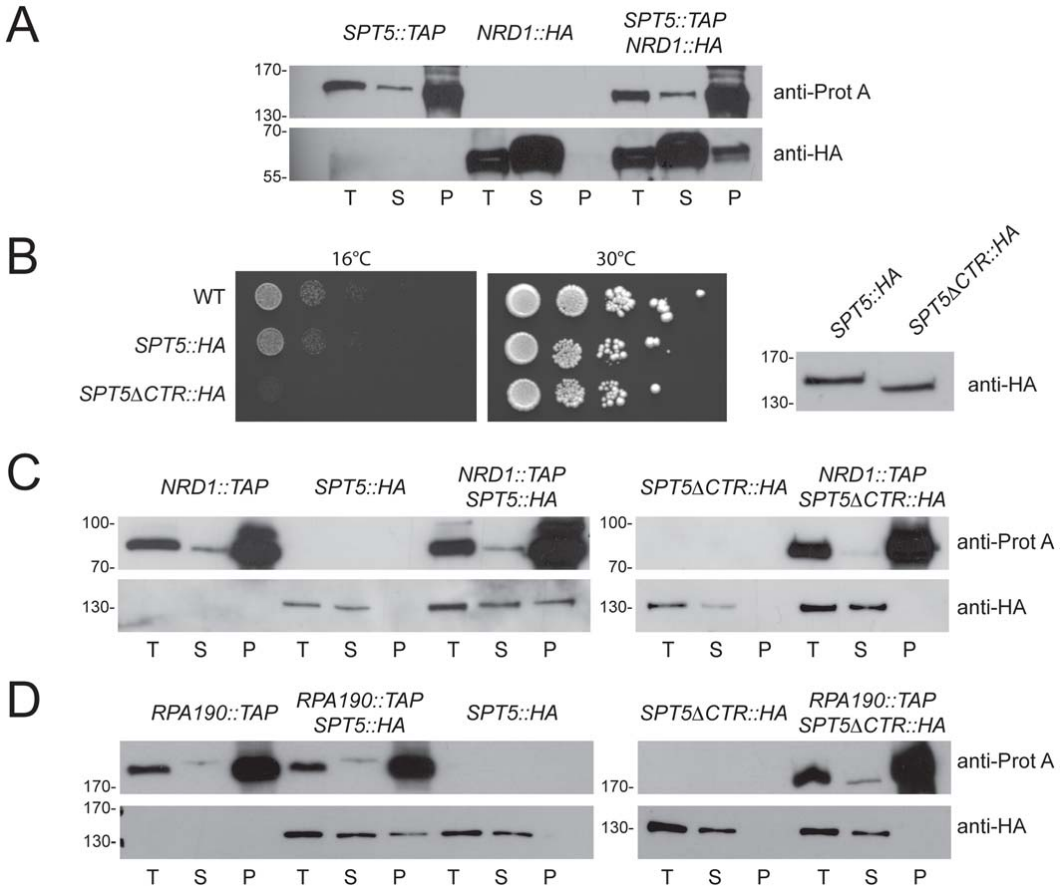
Spt5 interacts with Nrd1 and with the RNA Pol I through its conserved CTR. **A,** Spt5 interacts *in vivo* with Nrd1 Cells expressing functional Spt5-TAP and Nrd1-HA fusions were grown to mid-log phase in complete medium at 30°C. Glass-bead lysates were used in affinity purification analysis with IgG-coated magnetic beads that specifically capture TAP-tagged constructs. Isogenic strains expressing Spt5-TAP or Nrd1-HA alone were used as controls. Fractions of the total (T), supernantant (S) and pellet (P) were loaded in a 1∶1∶20 ratio. Membranes were probed with peroxidase-anti-peroxidase raised in rabbit (recognizes the Protein A component of the TAP-tag fusions) or an anti-HA antibody. This experiment was repeated three times. **B**, Characterization of a *SPT5ΔCTR::HA* construct. The precise deletion of the CTR of Spt5 in the genome of haploid cells resulted in a cold-sensitive phenotype for growth (left, cells were incubated for 5 d at 16°C, 2 d at 30°C), and the expression of a protein reduced in length by the expected size (right). The presence of an HA-tag at the carboxyl end of the construct, which did not affect functionality of the full-length construct (see *SPT5::HA* at 30°C), allowed Western-blot detection with an anti-HA antibody. **C**, The interaction between Spt5 and Nrd1 requires the CTR domain of Spt5. Cells expressing functional Nrd1-TAP and either a full-length Spt5-HA fusion, or a Spt5-HA construct precisely truncated for its entire CTR, were used as described in panel A. This experiment was repeated two times. **D,** The interaction between Spt5 and RNA Pol I requires the CTR domain of Spt5. Cells expressing functional Rpa190-TAP and either a full-length Spt5-HA fusion, or a Spt5-HA construct truncated for its CTR were used as described in panel A. This experiment was repeated three times. To the left of each western blot panel, molecular weight markers (in kDa).

Experiments by Vasiljeva and Buratowski showed that Nrd1-Nab3 exist in a complex with RNA Pol II elongation factors ([21]). We first confirmed this interaction in a reciprocal targeted affinity purification analysis where a functional Spt5-TAP fusion was used as a bait in a strain expressing as its sole source of Nrd1 a carboxyl HA-tagged construct from the endogenous promoter (Fig 4A).

Spt5 carries a highly conserved repetitive carboxyl terminal region (CTR), consisting of 15 repeats of the hexamer S-T/A-W-G-G-A/Q, somewhat reminiscent of the RNA Pol II CTD ([33]). This domain was precisely deleted by homologous recombination, directly on the chromosome, resulting in a strain that was viable, in agreement with previous observations ([34], [35]), showed increased sensitivity to cold (see also [36] [15]), and expressed as its sole source of Spt5 a protein reduced in size by the expected length (Fig 4B). In this experiment, Spt5 was derivatized with a C-terminal HA tag, which allowed detection in Western blotting. We found that in the absence of its CTR domain, Spt5 no longer interacted with Nrd1 (Fig 4C).

Spt5 was reported to interact with RNA polymerase I subunits *in vivo* ([10], [14]). We confirmed that Spt5 interacts with Rpa190 by affinity purification in cells expressing functional carboxyl terminal epitope-tagged constructs of Rpa190 and Spt5 (-TAP and -HA, respectively). Further, we demonstrated that the CTR domain of Spt5 is also required for this interaction (Fig 4D). Note that the deletion of the Spt5 CTR did not appear to significantly affect the cellular levels of Nrd1-TAP, Rpa190-TAP or Spt5-HA.

### The Spt4-Spt5 complex is required for efficient pre-rRNA processing

**Figure 5:**
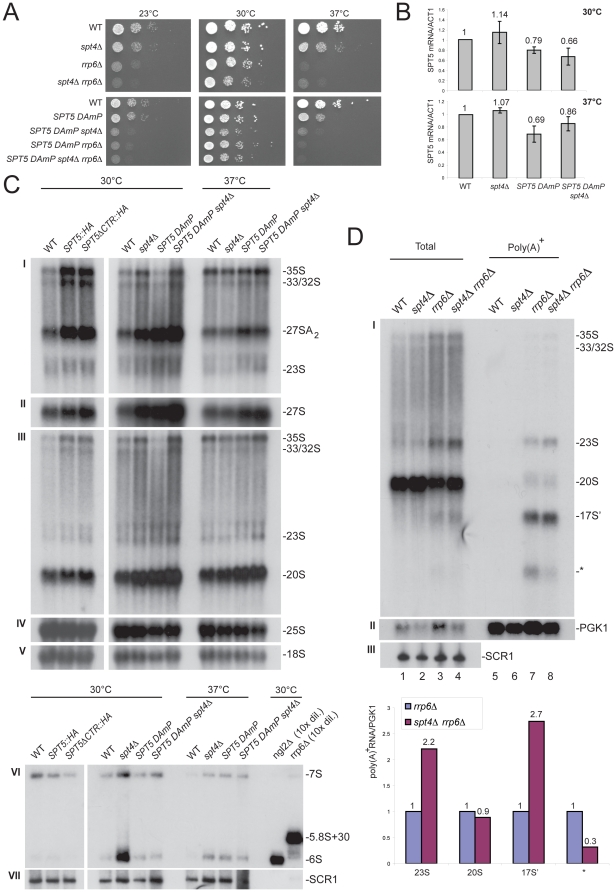
The Spt4-Spt5 complex interacts functionally with Rrp6, is required for efficient pre-rRNA processing, and is involved in nucleolar surveillance. A, Spt4 and Spt5 interact functionally with Rrp6. Legend as in Fig 1. B, RTqPCR analysis of the mRNA level of *SPT5* (normalized to ACT1) in *SPT5 DAmP* at different temperature in the presence, and in the absence of Spt4 (n = 3). C, Pre-rRNA processing analysis. Total RNA was extracted from different mutants grown at the indicated temperature, separated on denaturing gels. Probes used to detect pre-rRNAs and rRNAs were: LD359 (panel I), LD339 (panel II), LD471 (panel III), LD1099 (panel IV), LD871 (panel V), and LD906 (panel VI); they are depicted in Fig S3A. Panel VI, as a control for detection of 6S and 5.8S+30, total RNA was extracted from *ngl2* and *rrp6* mutants, respectively. These precursors are so abundant in these mutants, that 10x less RNA was loaded in these lanes; consequently, these lanes show no signal for SCR1 on this exposure. This experiment was repeated three times and a representative case is shown. D, Spt4 is involved in nucleolar surveillance. (top) Northern-blot analysis of total and purified poly(A)^+^ RNAs in different mutant background at 30°C (see legends to Figs 1 and 2). (bottom) Phosphor Imager quantitation (see legends to Figs 1 and 2).

To strengthen the view that RNA synthesis and nucleolar surveillance are connected, we established, using a growth plate assay, that the Spt4-Spt5 complex interacts functionally with Rrp6 by combining mutations in each genes (Fig 5A). For the essential *SPT5* gene, we made use of an hypomorphic DAmP allele (*D*ecreased *A*bundance by *m*RNA *P*erturbation, [37]). In the *SPT5DAmP* allele, the level of *SPT5* mRNA is decreased between 20 and 30%, depending on growth conditions (30°C versus 37°C) and whether Spt4 is present or not (Fig 5B).

Previous analysis established that cells deleted for *SPT4,* or expressing point mutations in Spt5 that only partially affect its function, show altered pre-rRNA kinetics ([10], [12]), we were therefore interested to establish the steady-state accumulation of precursor and mature rRNAs in various *spt* mutants. The *spt4*Δ and *spt5DAmP* alleles were expressed individually, or in combination, and total RNA was extracted from cells grown either at 30°C or 37°C, a temperature at which the growth defects were all exacerbated (see panel A). In addition, cells expressing a version of Spt5 truncated of its CTR were analyzed. Analysis of RNA steady-state levels indicated that strains defective for either component of the Spt4-Spt5 complex accumulated most of the pre-rRNA intermediates, starting with the accumulation of the primary transcript (35S) and early nucleolar precursors (33/32S) (Figs 5C, S3 and S4). The most striking accumulation was for the 20S and 27SA_2_ pre-rRNAs resulting from cleavage of the 32S pre-rRNA at site A_2_, located near the mid-point of ITS1, and destined to the small and large subunits, respectively (see Fig S3). At 37°C, these rRNA precursors were also accumulated, albeit at a lower level, possibly because at this temperature pre-rRNPs are less stable and favor RNA degradative pathways. Close inspection indicated that *spt4* and *spt5* mutants were also marginally affected for ITS2 processing (6S and 7S accumulation).

Altogether, these observations are compatible with the notion that in *spt4* and *spt5* mutants there is a disconnection between the pre-rRNA processing and surveillance machineries leading to some stabilization of the RNAs. This view is further supported by the observation that deletion of *SPT4* enhances the accumulation of several polyadenylated pre-rRNA precursors detected in *rrp6* mutants (Fig 5D).

## Discussion

In this work, we were interested in characterizing the physical and functional interface lying at the rDNA locus between the rRNA synthesis, processing and surveillance activities. Previous analysis by the teams of Profs Nomura and Schneider established that rRNA synthesis and pre-rRNA processing are functionally connected and that there are commonalities in the process of transcription elongation by the RNA Pol I and II. The Spt4-Spt5 complex, in particular, was shown to be a shared *trans*-acting factor (see Introduction). Nucleolar surveillance comprises both 5′-3′ and 3′-5′ pathways. Here, we have focused on the 3′-5′ pathway, which involves the addition of short poly(A) tails at the 3′-ends of unfaithful rRNA transcripts by TRAMP complexes, followed by complete digestion by the exosome.

**Figure 6:**
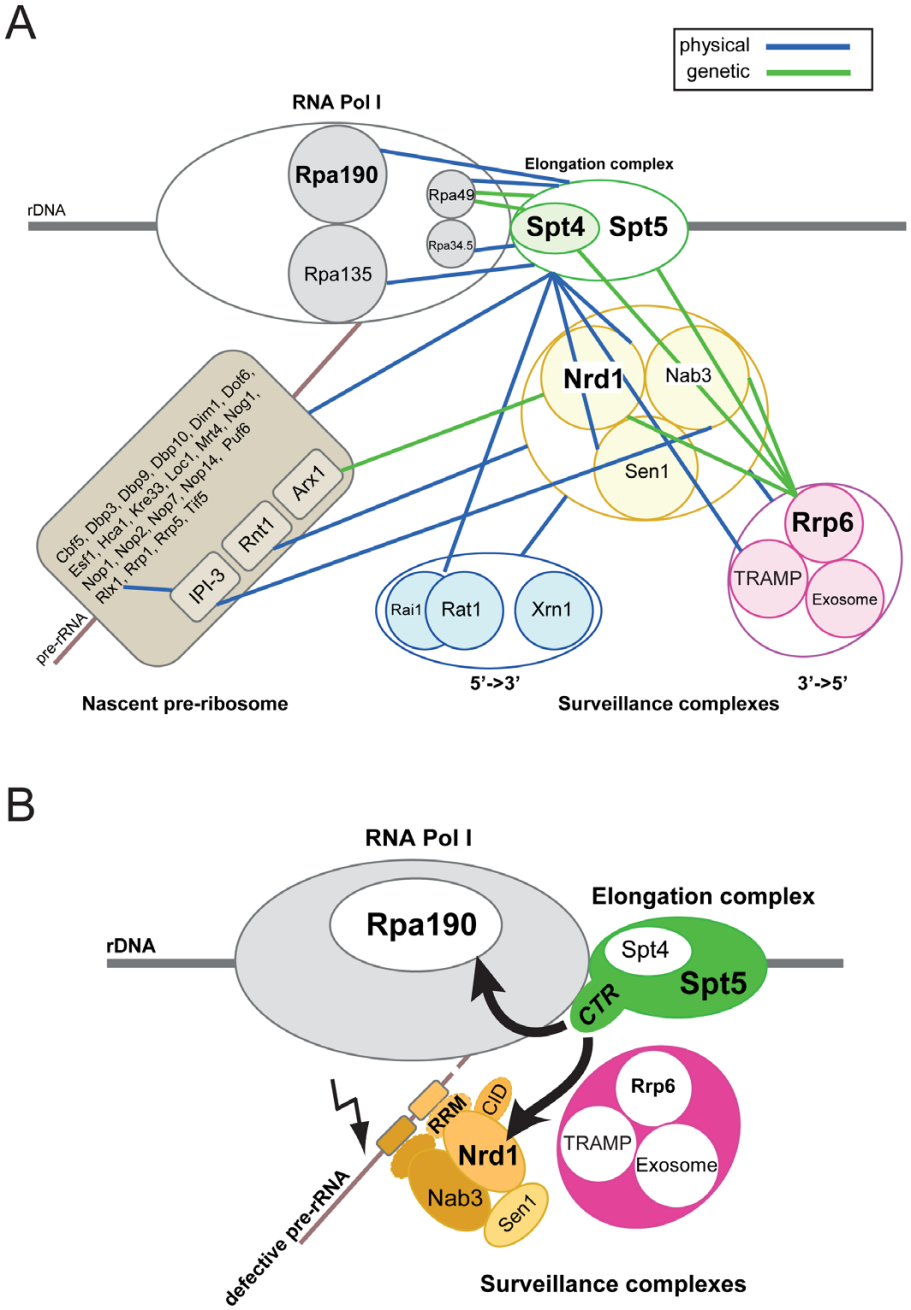
A physico-functional interface at the rDNA that connects rRNA synthesis, rRNA processing, and rRNA surveillance. **A,** The RNA Pol I, the Spt4–Spt5 elongation complex, the Nrd1-Nab3-Sen1, and the RNA exosome interact physically and are poised to interact functionally. We suggest that the Nrd1-Nab3-Sen1 complex contributes to recruiting 3′-to-5′ nucleolar surveillance to nascent pre-rRNA transcripts. Interactomics suggest that 5′-3′ nucleolar surveillance might also be recruited, but this has not addressed in this work. Rpa135-Rpa190, catalytic core of RNA Pol I; Rpa34.5-Rpa49, intrinsic elongation factor. Color-code: blue (physical interaction, affinity purification and *in vitro* pull-down), green (genetic interaction, limited to synthetic growth defect). Interaction dataset curated from BioGrid and from this work. **B,** Model for early recognition of defective pre-ribosomes: in conditions in which ribosome assembly is compromised (delayed assembly of ribosome synthesis factors or ribosomal proteins and/or misfolded RNA, lightning symbol), we speculate that cryptic Nrd1-Nab3 binding sites become available for binding. We suggest that the elongation factor Spt4–Spt5 recruits and deposits the Nrd1-Nab3 complex, which in turn attracts the RNA exosome to defective pre-rRNA transcripts.

Interactomics revealed an extensive network of physical interactions between Spt5 and numerous pre-ribosome constituents (Fig 6A), consistent with a function of the Spt4–Spt5 complex in ribosome synthesis. We have further characterized the function of the Spt4–Spt5 complex in ribosome synthesis and found that both proteins interact functionally with Rrp6 and that they are required for efficient pre-rRNA processing (Fig 5). The striking accumulation of pre-rRNA intermediates in *spt* mutants suggest that there is a disconnection between the pre-rRNA processing and surveillance activities leading to rRNA stabilization. We have confirmed by chromatin immunoprecipitation that the Spt4-Spt5 complex colocalizes across the RNA Pol I coding region (Fig 3). The interaction between Spt5 and the rDNA was reduced in the absence of Spt4.

The Spt4–Spt5 complex has been involved in regulating the processivity of RNA Pol II ([13], [38]), as well as in several transcription-coupled processes, such as nucleotide excision repair ([35]), pre-mRNA capping ([39]), pre-mRNA splicing ([14]) and mRNA localization ([40]). In addition, the complex has been shown to have a role in antibody diversification ([41]), vertebrate embryogenesis and neuronal development ([42], [43]), and viral gene expression (reviewed in [44]). The CTR domain of Spt5 consists of 15 repeats of a phosphorylated hexamer, somewhat reminiscent of the CTD of Rpb1, the largest subunit of RNA Pol II ([33]). We have confirmed that the RNA polymerase elongation factor Spt5 interacts *in vivo* with the largest subunit of RNA polymerase I and with the exosome cofactor Nrd1, and we have demonstrated that both of these interactions require the CTR domain of Spt5 (Fig 4). *In vitro*, Spt5 binds directly to RNA Pol I and RNA Pol II through its conserved central region ([15]). It is quite remarkable that while the CTR is dispensable for these interactions *in vitro* ([15]), we have found it to be necessary for the interaction between Spt5 and Rpa190 *in vivo* (Fig 3). This indicates that the association between Spt5 and RNA Pol I likely relies on a complex network of interactions whose disruption has distinctly different consequences *in vitro* and *in vivo*. Interestingly, the Spt5 CTR is not required for interaction with Rpb1 *in vivo* ([35]), thus our observation provides initial insights into discriminating the involvement of Spt5 in RNA Pol I and RNA Pol II function.

The Nrd1-Nab3-Sen1 complex is a sequence specific RNA binding complex that interacts physically and functionally (Figs 1 and 2) with the nuclear RNA exosome and whose function in the termination of short RNA Pol II transcripts has been extensively characterized (reviewed in [19], [20]). The association of the Nrd1-Nab3-Sen1 complex with the exosome allows the coupling of RNA transcription termination with either controlled RNA digestion, accompanied by the generation of mature 3′-termini (e.g. snoRNAs), or the complete degradation of the RNA (e.g. CUTs). The mechanism of termination of short noncoding RNA Pol II transcripts is not clear; it is thought, however, to occur in a cleavage-dependent fashion, although the nuclease responsible is not known. It often occurs just downstream of predicted Nrd1 and Nab3 binding sites and requires both additional *cis*-acting elements and a subset of proteins involved in the cleavage of polyadenylated mRNAs (discussed in [45]), neither of which is expected to be found in the Pol I context. We have shown that the Nrd1-Nab3 complex colocalizes with the RNA Pol I coding region (Fig 3), with a striking enrichment towards the 3′-end of the gene suggesting that it might carry additional function in RNA Pol I transcription termination. A known interacting partner, Sen1, was previously shown to localize at the rDNA ([46]). RNA Pol II transcription is known to occur at the vicinity of the rDNA coding regions, in the IGSs ([27], [47]), producing unstable transcripts; this transcription likely contributes to the local enrichment of surveillance factors and of other shared *trans*-acting factors. We have demonstrated in *rrp6Δ* cells that the inactivation of Nrd1 leads to a 3-4 fold accumulation of normal pre-rRNA intermediates (e.g. 20S pre-rRNA) and aberrant truncated rRNA transcripts (e.g. 17S' and *), known to be polyadenylated by TRAMP5 ([6]) and destined to be degraded by Rrp6 (Fig 1). Importantly, stabilization of rRNA transcripts was only observed upon inactivation of the RRM of Nrd1, not when its CID was compromised. Inactivation of the RRM domain of Nab3 also led to some stabilization of 17S' polyadenylated rRNA strengthening the role of the Nrd1 complex in this surveillance (Fig 2). The CID domain of Nrd1 is known to specifically recognize the Ser5-phosphorylated form of the Pol II CTD ([29]). This indicates that rRNA stabilization is not dependent upon the interaction of Nrd1 with the RNA Pol II CTD. Again, this observation provides initial insights into discriminating the involvement of shared *trans*-acting factors in RNA Pol I and RNA Pol II function.

Our dataset is compatible with a model in which Nrd1 contributes to recruiting the 3′-5′ nucleolar surveillance to nascent pre-rRNAs through an interaction with the RNA elongation factor Spt5 (Fig 6B). Our suggestion is that in conditions in which ribosome assembly is compromised, some of the numerous cryptic Nrd1 and Nab3 consensus sites present on nascent pre-rRNAs, which we believe are otherwise rapidly covered by ribosomal proteins and ribosome synthesis factors, and buried within the highly structured pre-rRNAs, rendering them inaccessible, become available for binding by the Nrd1-Nab3-Sen1 complex. Quite similarly, it was suggested recently that the expression of the bacterial termination factor Rho in yeast cells ‘competes out’ and displaces normal mRNA processing and packaging factors from pre-mRNAs, making cryptic Nrd1 binding sites available, and leading to the recruitment of surveillance activities ([48]). In a recent genome-wide screening aimed at identifying Nrd1 and Nab3 binding sites by UV-cross linking and high-throughput sequencing of the captured RNAs, many hits were recovered in oligoadenylated Pol III transcripts, predominantly pre-tRNAs, and the depletion of Nrd1 or Nab3 was shown to stabilize tested Pol III transcripts ([22]). These observations are consistent with the model discussed here that the Nrd1-Nab3 complex carries Pol II-independent functions.

By analogy to the mode of action of the Nrd1-Nab3-Sen1 complex in the termination of short RNA Pol II transcripts, that is, the recruitment of Nrd1 at the Ser5-phosphorylated CTD that predominates at the 5′-end of genes, and the cooperative deposition of Nrd1-Nab3 complexes on nascent transcripts carrying consensus protein binding sites, we speculate that a function provided in *cis*- by the RNA Pol II CTD is alternatively provided in *trans*- by the CTR of Spt5.

In mammals, the Spt5 CTR is phosphorylated by Cdk9/P-TEFb, and this is instrumental in converting DSIF (human Spt4–Spt5) from a repressor to a positive regulator of transcription ([49], [50]). In yeast, the Spt5 CTR is phosphorylated by the BUR (Bur1-Bur2) kinases ([34], [35], [36]). Future work will establish whether the phosphorylation component of the Spt5 CTR is required for its interaction with the ribosomal RNA synthesis and surveillance activities.

While several ribosome surveillance mechanisms have recently been described, an outstanding question in the field has remained to understand how defective ribosomes are recognized. Our model postulates that the Nrd1-Nab3 complex contributes to selecting unfaithful pre-ribosomes by binding to cryptic sites on nascent pre-rRNA transcripts and recruiting TRAMP and the RNA exosome.

## Materials and Methods

### Yeast genetics

Yeast cells were grown according to standard procedures in YPD (2% peptone, 1% yeast extract, 2% glucose). The *nrd1-101, nrd1-102* and *nab3-11* temperature-sensitive alleles have been described ([51]; [24]; [25]). Temperature shifts were performed at 37°C for 2 h. Cultures were kept in mid-log phase at all times by continuous dilution with fresh medium. The yeast strains, and the oligonucleotides used to construct them, are listed on-line in Materials and Methods S1.

### Chromatin Immunoprecipitation

Yeast cells were grown in 25 ml YPD at the appropriate temperature to OD_600_ 0.8, fixed with 750 µl of 37% formaldehyde (Sigma) for 15 min at 25°C. For the Spt4–Spt5 complex, and respective controls, cells were fixed for 20 min, as described in [10]. The cross-link reaction was stopped by adding 5 ml of 2.5 M glycine for 5 min at 25°C. Fixed cells were harvested by centrifugation, washed in 10 ml ice-cold 10 mM Tris-HCl pH 8.0, and stored at −80°C. Cells lysis was performed with glass beads in FA/SDS/PMSF buffer (50 mM Hepes–KOH pH 7.5, 150 mM NaCl, 1 mM EDTA, 1% Triton X100, 0.1% sodium deoxycholate, 0.1% SDS, and 1 mM PMSF) by vortexing for 20 min at 4°C. The cross-linked material was harvested by centrifugation at 12 krpm for 35 min at 4°C, and the supernatant discarded. Chromatin was washed in 1.6 ml FA/SDS/PMSF buffer for 1 h at 4°C, centrifuged at 13.2 krpm for 20 min at 4°C, and resuspended in 300 µl FA/SDS/PMSF buffer. The chromatin was sonicated in a bioruptor UDC200 (Diagenode, setup high for 10 min, with cycles of 30 s on/1 min off) to yield DNA fragments with an average size of 500 bp. 1.3 ml of FA/SDS/PMSF was added prior to a final centrifugation at 10 krpm for 45 min at 4°C. The supernatant was stored at −80°C. Immuno-precipitation was performed starting with 500 µl of chromatin, for 2 h at 21°C and at 1.3 krpm in a Thermomixer (Eppendorf). The TAP-tagged proteins were directly coprecipitated with the Pan Mouse IgG Dynabeads ([52]). For further details see [6]. Biological triplicates (i.e. three independent cultures) were generated for each experiment. DNA material in the IP and Input preparations was quantified by real-time PCR (qPCR) using the Platinum SYBR Green qPCR SuperMix-UDG with Rox (Invitrogen) on Applied Biosystems hardware. IP data were normalized to the input. Primer sequences for qPCR are indicated in Materials and Methods S1. p-values were calculated with the software GraphPad prism v5.

### Western blotting

Total protein was extracted from 20 cell OD_600._ Total RNA (Figs 1 and 4 and S2) and total protein (Figs 3B, S2B–C) were extracted from the same cultures. Cells lysis was performed with glass beads in 100 µl IP buffer (see below) by vortexing 5 times 1 min, with 30 s intervals on ice. The supernantant was recovered following a 15 min centrifugation at 13.2 krpm. Samples were resuspended in SDS loading buffer (100 mM TrisCl pH 6.8, 4% SDS, 0.2% BPB, 20 mg glycerol, 100 mM DTT, 2.5%ß-mercaptoethanol), loaded on 10–12% SDS-PAGE, and transferred to PVDF (GE Healthcare). Membranes were blocked for 1 h in 1x PBS-3% milk and either incubated with a rabbit anti-Protein A (P-3775, Sigma, 1∶2000, 2 h), or with a mouse anti-HA (MMS-101P, Covance, 1∶1000, 2 h) followed by an anti-mouse-HRP (Sc-2005, Santa Cruz biotechnology, 1∶5000, 1 h). The pageRuler^TM^ prestained protein ladder (Fermentas, SM0671) was used to confirm that the fusions had the expected size. For ChIP control, primary antibodies were used at 1∶1000 dilutions in PBS-3% milk and revealed with the supersignal west pico chemiluminescent kit (Pierce).

### RNA extraction and Northern blotting

RNA extraction and Northern blotting were as described previously in [53]. Oligonucleotides used as probes to detect rRNA are listed in Materials and Methods S1. Total RNA and poly(A)^+^ RNA were either analyzed on 1.2% agarose/6% formaldehyde and 6–8% acrylamide-urea gels. Oligonucleotides used in the hybridizations are listed in Materials and Methods S1. For the detection of unstable readthrough transcripts, 10 µg of total RNA was resolved on a 2% agarose/6% formaldehyde gel and transferred to nylon. The Northern-blot membrane was hybridized with a T7 RNA probe, which was prepared as follows: a DNA template was amplified by PCR with oligonucleotides containing the sequence of a T7 RNA polymerase promoter (see Materials and Methods S1). 150 ng of the resulting PCR product was used as a DNA template and added to a reaction mix containing 2 µl of transcription buffer 10x, 1 µl of T7 RNA polymerase (Roche), 100mM DTT, 2mM each of ATP, CTP and GTP (Roche), rRNasin (Promega), and alpha-P32 UTP (Perkin Elmer) in a volume of 20 µl and incubated at 37°C for 30 min. The hybridization was performed in UltraHyb buffer (Ambion) overnight at 68°C.

### Poly(A)^+^ RNA purification

Poly(A)^+^ RNA purification used 500 µg of total RNAs and the Poly(A)tract mRNA isolation system IV (Promega).

### RNA quantitation

All quantitations were performed on a Fuji phosphoimager FLA-7000 and used the Multi Gauge v3.1 native software.

### Affinity purifications

Yeast cells expressing suitable epitope-tagged constructs were grown in YPD at 30°C and 250 ml were harvested at OD_600_ 0.5. The yeast cells were lysed with glass beads and total proteins was extracted with 1 ml of IP buffer (20 mM HEPES pH7.5, EDTA, 100 mM NaCl, 20% glycerol, 0.05% NP-40, 2.5 mM PMSF, 1 mM DTT). Immuno-precipitation was performed with 400 µl of total protein and 40 µl of IgG-coated magnetic beads (Dynabeads Pan mouse IgG, Invitrogen), previously washed three times with PBS-0.1% BSA and resuspended in PBS-1% BSA, for 2 h at 21°C and at 1.3 krpm in a Thermomixer (Eppendorf). The beads were washed once with 400 µl IP buffer and three times with IP buffer supplemented with 150 mM NaCl. The elution was performed for 20 min at 65°C in elution buffer (125 mM Tris pH7.5, 25 mM EDTA, 2.5% SDS).

### RTqPCR analysis

Reverse transcription reactions were performed with Superscript II (Invitrogen), according to the supplier's instructions, using 1 µg of total RNA previously treated by DNase I (Fermentas). Real-time PCR used the Platinum SYBR Green qPCR SuperMix-UDG with Rox (Invitrogen) on Applied Biosystems hardware. The qPCR datasets were analyzed using the ΔΔCt method. All results were normalized to ACT1. Primer sequences for PCR are provided on-line in Materials and Methods S1.

## Supporting Information

Figure S1 **Mapping Nrd1 and Nab3 consensus binding sites bioinformatically onto yeast pre-rRNA.** The sequence encoding the large RNA Pol I transcript in budding yeast was analyzed for the presence of Nab3 and Nrd1 consensus binding motifs with a dedicated software package (available at http://rsat.bigre.ulb.ac.be/rsat/, [54]). Consensus motifs for Nab3 and Nrd1 binding sites are short, making them ubiquitous in large RNAs. To increase the possibility of attaining biologically relevant hits, the yeast sequence was randomized, and two additional rRNA sequences from genomes that do not encode orthologs of Nab3 and Nrd1 were used (*E. coli* and *A. sulfolobus*). Randomizing the yeast rRNA sequence significantly reduced the number of hits for Nab3 binding sites and had no effect on the number of Nrd1 hits. There were three times less Nab3 hits in bacterial and archaeal sequences than in yeast; the number of putative Nrd1 sites was unaffected.
(TIF)

Figure S2 **The thermoinactivation of Nrd1 or Nab3 leads to** ***GRE1*** **readthrough transcript stabilization. A,** Genomic structure of the locus used for readthrough transcript detection. The enlarged open arrow represents a cryptic unstable transcript (CUT) promoter, the closed arrows are promoters of adjacent genes (see [51]). Transcripts are depicted. **B,** *nrd1-102* analysis. (left) Validation of the *nrd1* inactivation by detection of 3′-extended readthrough products. Readthrough transcripts are not normally detected in wild-type strains but they are stabilized upon exosome inactivation (lanes 5–6) or when their termination is compromised (lanes 3–4, see [51]). The concomitant inactivation of *RRP6* and *NRD1* leads to a strong synergistic effect on readthrough transcript stabilization. Note that upon inactivation of both *NRD1* and *RRP6*, an additional, shorter band (GRE1*) is stabilized. (right) Western blot analysis of Nrd1-HA steady-state accumulation in different mutants grown at different temperatures. Total protein was extracted from the same cultures as those used in the RNA analysis presented in Fig 1. The Western blot was probed with an anti-HA antibody. **C,** *nrd1-101* analysis. Legend as in panel B. **D,** *nab3-11* analysis. Legend as in panel A. **E,** Legend as in panel B.
(TIF)

Figure S3 **Yeast pre-rRNA processing pathway. A,** rDNA unit and probes used in this work. A single large RNA Pol I transcript (35S) encodes three out of the four ribosomal RNAs. The coding sequences for the 18S, 5.8S and 25S rRNAs are flanked by the 5′- and 3′-external (5′- and 3′-ETS) and internal transcribed spacers 1 and 2 (ITS1 and ITS2). Cleavage sites (A_0_ to E) and the oligonucleotides, used in the Northern-blot hybridizations are indicated. The fourth rRNA (5S) is synthesized independently by RNA Pol III (not represented). **B,** Pre-rRNA processing pathway. The 35S RNA is initially cleaved at sites A_0_–A_2_ by the SSU-processome. The resulting 20S and 27SA_2_ pre-rRNAs are destined to the small and large subunit, respectively. The 20S pre-rRNA is exported to the cytoplasm where it is converted into 18S rRNA, following 3′-end endonucleolytic cleavage at site D by Nob1. The 27SA_2_ pre-rRNA is matured following two alternatives pathways resulting in the production of two forms (short and long) of 5.8S rRNA that differ in size by about 7 nucleotides at their 5′-ends. In the major pathway (representing ≈80% of molecules), 27SA_2_ is endonucleolytically cleaved at site A_3_ by RNase MRP, and digested to site B_1S_ by the exoRNases Rat1-Rai1 and Rrp17. In the minor pathway (≈20%), the 27SA_2_ is cleaved endonucleolytically at site B_1L_ by an unknown activity. Both forms of 27SB pre-rRNAs are cleaved at site C_2_ within ITS2, generating the 7S pre-rRNAs, precursors to the 5.8S, and the 26S pre-rRNA, precursor to the 25S rRNA. The 7S pre-rRNA is digested to site E, corresponding to the 3′-end of 5.8S, by an extremely complex succession of reactions involving the core exosome, the nuclear specific subunit exosome sununit Rrp6, Ngl2 and the Rex exoRNases. The final steps of 5.8S 3′-end formation occurs in the cytoplasm. The 26S pre-rRNA is digested to site C_1_, the 5′-end of the 25S rRNA, by Rat1. In fast growing cells, up to 70% of transcripts are cleaved cotranscriptionally in ITS1 (not represented). **C,** Aberrant pre-rRNA precursors. In normal conditions, cleavage at sites A_0_–A_2_ is tightly coupled and largely occurs prior to cleavage at site A_3_. Under perturbed conditions, pre-rRNA processing kinetics might be altered, leading to premature cleavage at A_3_ and partial or complete uncoupling of cleavages at A_0_–A_2_. As a result, aberrant RNAs, such as the 21S and 23S, are generated. The 23S extends from the transcription start site to A_3_. The 21S RNA extends from site A_1_ to A_3_. The 17S', previously described in [6], [26], extends from position +1100/+1150 (data not shown), with respect to the transcription start site, to site A_3_. How the 5′-end of 17S' is generated is currently unclear. One possibility is that it corresponds to a strong secondary structure that impedes exoribonucleolytic digestion; alternatively it might directly result from an endonucleolytic cleavage at a cryptic site by salient cellular RNases.
(TIF)

Figure S4 **Quantitation of RNA ratio of Northern blots presented in** **Fig 5C** **.**
(TIF)

Materials and Methods S1 **Strains and oligonucleotides used in this study.**
(DOC)
