# Supplementary figures and images for: A Functional Interface at the rDNA Connects rRNA Synthesis, Pre-rRNA Processing and Nucleolar Surveillance in Budding Yeast
Source: PLoS One. 2011 Sep 19;6(9):e24962. doi: 10.1371/journal.pone.0024962 (PMC3176313; doi:10.1371/journal.pone.0024962)

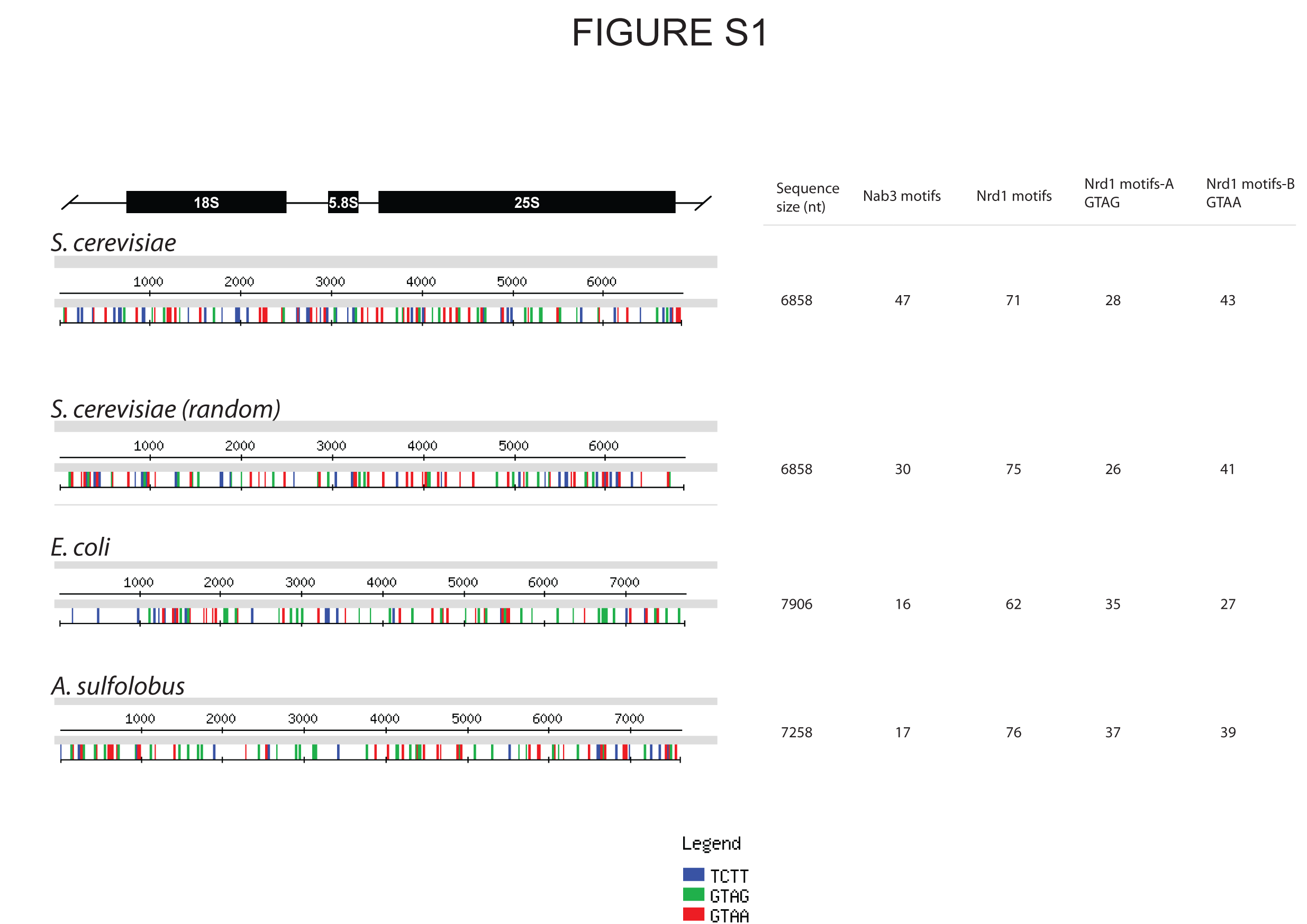

Supplement: Figure S1 — Mapping Nrd1 and Nab3 consensus binding sites bioinformatically onto yeast pre-rRNA. The sequence encoding the large RNA Pol I transcript in budding yeast was analyzed for the presence of Nab3 and Nrd1 consensus binding motifs with a dedicated software package (available at http://rsat.bigre.ulb.ac.be/rsat/, [54]). Consensus motifs for Nab3 and Nrd1 binding sites are short, making them ubiquitous in large RNAs. To increase the possibility of attaining biologically relevant hits, the yeast sequence was randomized, and two additional rRNA sequences from genomes that do not encode orthologs of Nab3 and Nrd1 were used (E. coli and A. sulfolobus). Randomizing the yeast rRNA sequence significantly reduced the number of hits for Nab3 binding sites and had no effect on the number of Nrd1 hits. There were three times less Nab3 hits in bacterial and archaeal sequences than in yeast; the number of putative Nrd1 sites was unaffected. (TIF) [file pone.0024962.s001.tif]

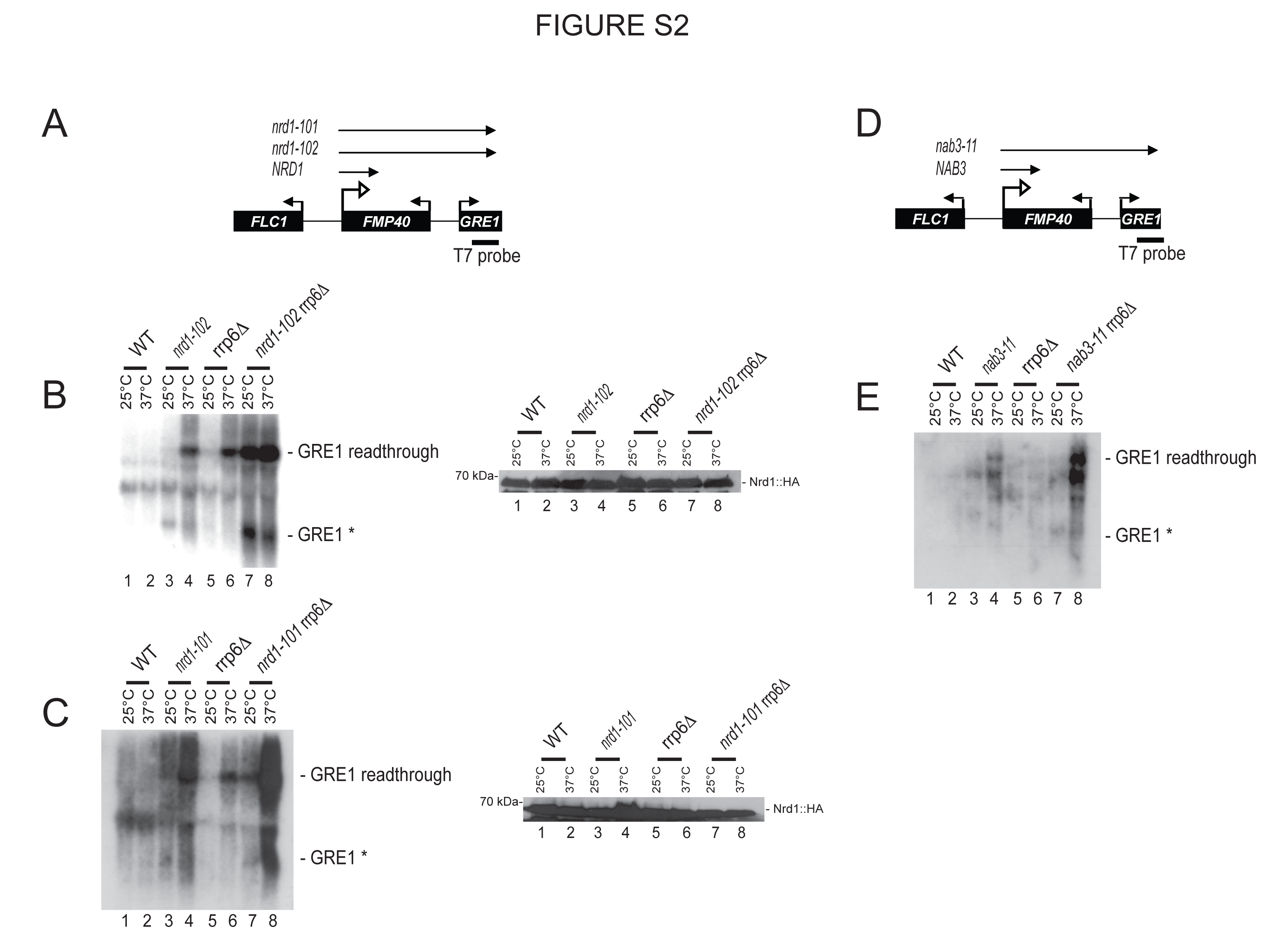

Supplement: Figure S2 — The thermoinactivation of Nrd1 or Nab3 leads to GRE1 readthrough transcript stabilization. A, Genomic structure of the locus used for readthrough transcript detection. The enlarged open arrow represents a cryptic unstable transcript (CUT) promoter, the closed arrows are promoters of adjacent genes (see [51]). Transcripts are depicted. B, nrd1-102 analysis. (left) Validation of the nrd1 inactivation by detection of 3′-extended readthrough products. Readthrough transcripts are not normally detected in wild-type strains but they are stabilized upon exosome inactivation (lanes 5–6) or when their termination is compromised (lanes 3–4, see [51]). The concomitant inactivation of RRP6 and NRD1 leads to a strong synergistic effect on readthrough transcript stabilization. Note that upon inactivation of both NRD1 and RRP6, an additional, shorter band (GRE1*) is stabilized. (right) Western blot analysis of Nrd1-HA steady-state accumulation in different mutants grown at different temperatures. Total protein was extracted from the same cultures as those used in the RNA analysis presented in Fig 1. The Western blot was probed with an anti-HA antibody. C, nrd1-101 analysis. Legend as in panel B. D, nab3-11 analysis. Legend as in panel A. E, Legend as in panel B. (TIF) [file pone.0024962.s002.tif]

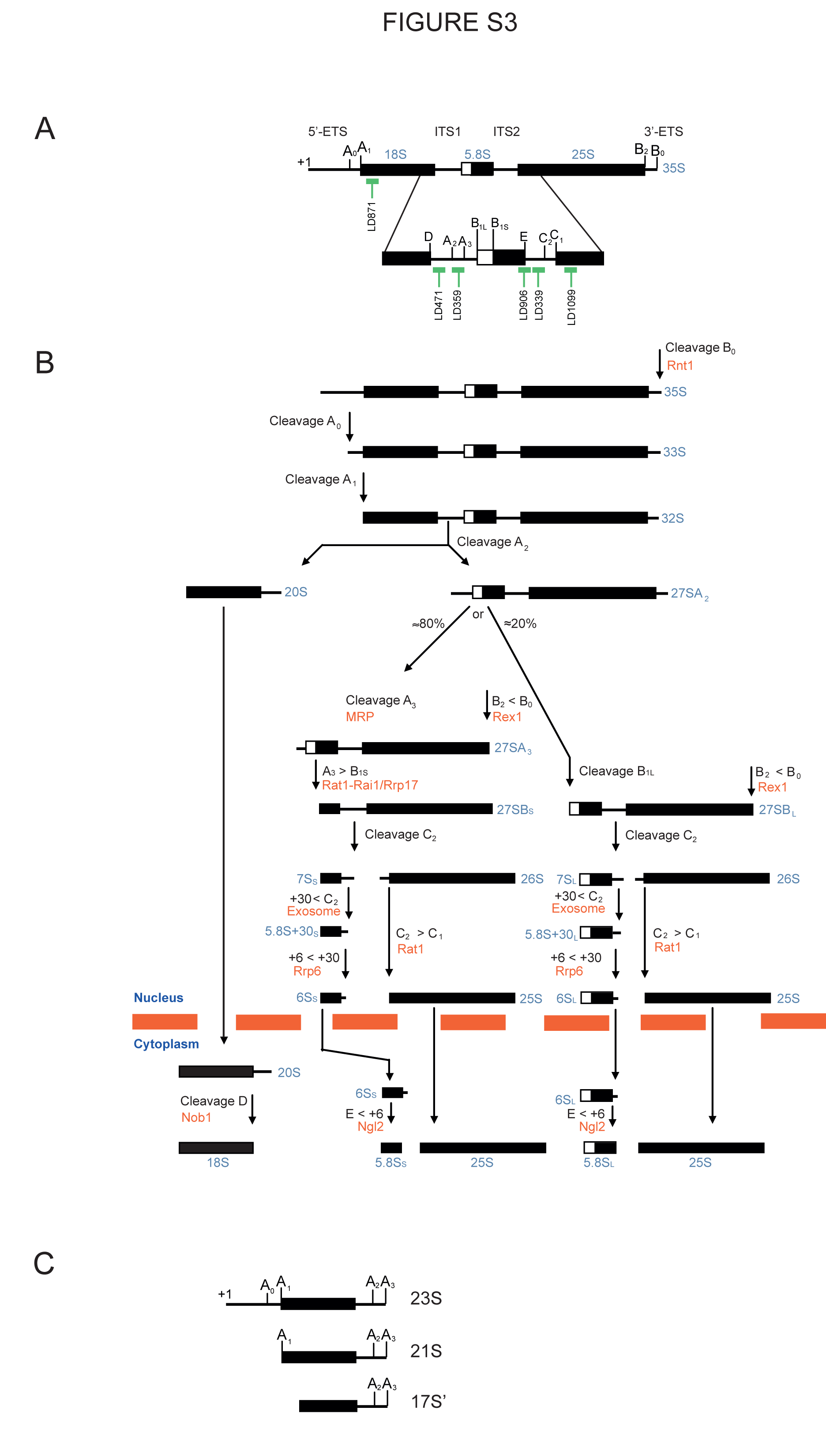

Supplement: Figure S3 — Yeast pre-rRNA processing pathway. A, rDNA unit and probes used in this work. A single large RNA Pol I transcript (35S) encodes three out of the four ribosomal RNAs. The coding sequences for the 18S, 5.8S and 25S rRNAs are flanked by the 5′- and 3′-external (5′- and 3′-ETS) and internal transcribed spacers 1 and 2 (ITS1 and ITS2). Cleavage sites (A0 to E) and the oligonucleotides, used in the Northern-blot hybridizations are indicated. The fourth rRNA (5S) is synthesized independently by RNA Pol III (not represented). B, Pre-rRNA processing pathway. The 35S RNA is initially cleaved at sites A0–A2 by the SSU-processome. The resulting 20S and 27SA2 pre-rRNAs are destined to the small and large subunit, respectively. The 20S pre-rRNA is exported to the cytoplasm where it is converted into 18S rRNA, following 3′-end endonucleolytic cleavage at site D by Nob1. The 27SA2 pre-rRNA is matured following two alternatives pathways resulting in the production of two forms (short and long) of 5.8S rRNA that differ in size by about 7 nucleotides at their 5′-ends. In the major pathway (representing ≈80% of molecules), 27SA2 is endonucleolytically cleaved at site A3 by RNase MRP, and digested to site B1S by the exoRNases Rat1-Rai1 and Rrp17. In the minor pathway (≈20%), the 27SA2 is cleaved endonucleolytically at site B1L by an unknown activity. Both forms of 27SB pre-rRNAs are cleaved at site C2 within ITS2, generating the 7S pre-rRNAs, precursors to the 5.8S, and the 26S pre-rRNA, precursor to the 25S rRNA. The 7S pre-rRNA is digested to site E, corresponding to the 3′-end of 5.8S, by an extremely complex succession of reactions involving the core exosome, the nuclear specific subunit exosome sununit Rrp6, Ngl2 and the Rex exoRNases. The final steps of 5.8S 3′-end formation occurs in the cytoplasm. The 26S pre-rRNA is digested to site C1, the 5′-end of the 25S rRNA, by Rat1. In fast growing cells, up to 70% of transcripts are cleaved cotranscriptionally in ITS1 (not represented). [file pone.0024962.s003.tif]

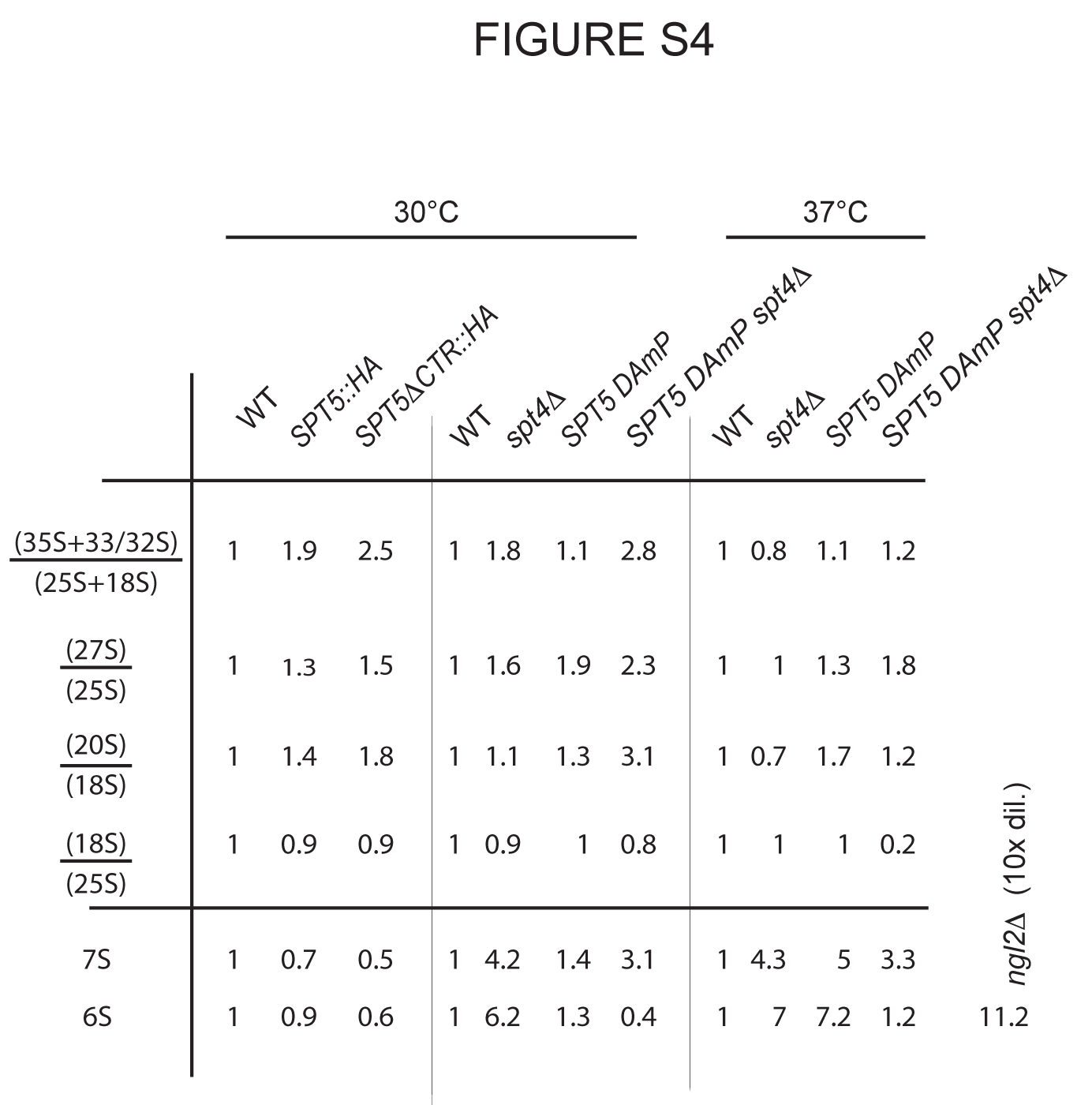

Supplement: Figure S4 — Quantitation of RNA ratio of Northern blots presented in Fig 5C . (TIF) [file pone.0024962.s004.tif]
