# Supplementary material for: A Functional Interface at the rDNA Connects rRNA Synthesis, Pre-rRNA Processing and Nucleolar Surveillance in Budding Yeast
Source: PLoS One. 2011 Sep 19;6(9):e24962. doi: 10.1371/journal.pone.0024962 (PMC3176313; doi:10.1371/journal.pone.0024962)
Supplement: Materials and Methods S1 — Strains and oligonucleotides used in this study. (DOC) [file pone.0024962.s005.doc]

**SUPPLEMENTAL MATERIALS AND METHODS**

**Yeast strains**

All strains were diagnosed by PCR on genomic DNA and, where relevant, by Western blotting, biochemical, and functional analysis. Strains were either purchased from Euroscarf, Open Biosystems, or purposely made by homologous recombination directly on the chromosome, following transformation of suitable PCR cassettes generated with oligonucleotides listed below. Some mutant strains are the generous gift from the Corden and Greenblat laboratories.

| **Strain name** | **Pet Name** | **Genotype** | **Oligonucleotides, DNA templates or plasmids used for construction** | **Host Strain** | **References** |
| --- | --- | --- | --- | --- | --- |
| YDL674 | WT | *Mat a his3∆1 leu2∆0 met15∆0 ura3∆0* |  |  | Euroscarf |
| YL1776 | *rrp6∆* | *Mat a his3∆1 [leu2∆0] met15∆0 ura3∆0 rrp6∆::CgLEU2* | PCR amplification from pBS1763 using oligonucleotides LD1222 and LD1223 | YDL674 | This work |
| YDL1678 alias YJC1163 | *NRD1::HA* | *Mat a his3∆1 leu2∆0 met15∆0 [ura3∆0] NRD1::HA* |  |  | [1,2] |
| YDL1782 | *NRD1::HA rrp6∆* | *Mat a his3∆1 [leu2∆0] met15∆0 [ura3∆0] NRD1::HA rrp6∆::CgHIS3* | PCR amplification from pBS1763 using oligonucleotides LD1222 and LD1223 | YDL1678 | This work |
| YDL1679  alias YJC1166 | *nrd1-102 ::HA* | *Mat a his3∆1 leu2∆0 met15∆0 [ura3∆0] nrd1-102::HA* |  |  | [1,2] |
| YDL1784 | *nrd1-102 ::HA rrp6∆* | *Mat a his3∆1 [leu2∆0] met15∆0 [ura3∆0] nrd1-102::HA rrp6∆::CgHIS3* | PCR amplification from pBS1763 using oligonucleotides LD1222 and LD1223 | YDL1679 | This work |
| YDL2545  Alias YJC1103 | *nrd1-101 ::HA* | *Mat a ura3∆0 his3∆1 [leu2∆0] met15∆0 NRD1::kamMX (pJC951 LEU2 nrd1-101::HA)* |  |  | [1,2] |
| YDL2549 | *nrd1-101 rrp6∆* | *Mat a ura3∆0 [his3∆1] leu2∆0 met15∆0 NRD1::kamMX (pJC951 LEU2 nrd1-10 ::HA) rrp6∆::CgHIS3* | PCR amplification from pBS1762 using oligonucleotides LD1222x1223 | YDL2545 | This work |
| YDL1680 alias YJC1412 | WT | *Mat a ade2 can1-100 his3-11,15 leu2-3,112 trp1-1 ura3-1* |  |  | [1,2] |
| YDL1788 | *rrp6∆* | *Mat a ade2 can1-100 his3-11,15 [leu2-3,112] trp1-1 ura3-1 rrp6∆::CgLEU2* | PCR amplification from pBS1763 using oligonucleotides LD1222 and LD1223 | YDL1680 | This work |
| YDL1681 alias YJC1099 | *nab3-11* | *Mat a ade2 can1-100 his3-11,15 leu2-3,112 trp1-1 ura3-1 nab3-11* |  |  | [1,2] |
| YDL1791 | *nab3-11 rrp6∆* | *Mat a ade2 can1-100 his3-11,15 [leu2-3,112] trp1-1 ura3-1 rrp6∆::CgLEU2 nab3-11* | PCR amplification from pBS1763 using oligonucleotides LD1222 and LD1223 | YDL1681 | This work |
| YDL1570 | *NAB3::TAP* | *Mat a his3∆1 leu2∆0 met15∆0 ura3∆0 NAB3::TAP-HIS3MX6* |  |  | [3] - Open Biosystems |
| YDL1569 | *NRD1::TAP* | *Mat a his3∆1 leu2∆0 met15∆0 ura3∆0 NRD1::TAP-HIS3MX6* |  |  | [3] - Open Biosystems |
| YDL2560 | *spt4∆* | *Mat a [his3∆1] leu2∆0 met15∆0 ura3∆0 spt4∆::CgHIS3* | PCR amplification from pBS1762 plasmid using oligos LD1961 and LD1962 | YDL674 | This work |
| YDL2570 | *rrp6Δ spt4∆* | *Mat a [his3∆1] [leu2∆0] met15∆0 ura3∆0 spt4::CgHIS3 rrp6∆::CgLEU2* | PCR amplification from pBS1762 plasmid using oligos LD1961 and LD1962 | YDL1776 | This work |
| BY4741 | WT | *Mat a his3∆1 leu2∆0 ura3∆0 met15∆0* |  |  |  |
| YDL2537 | *SPT5 DAmP* | *Mat a his3∆1 leu2∆0 ura3∆0 met15∆0 SPT5 DAmP* |  |  | [4] |
| YDL2572 | *SPT5 DAmP spt4∆* | *Mat a [his3∆1] leu2∆0 ura3∆0 met15∆0 SPT5 DAmP spt4∆::CgHIS3* | PCR amplification from pBS1762 plasmid using oligos LD1961 and LD1962 | YDL2537 | This work |
| YDL2574 | *SPT5 DAmP rrp6∆* | *Mat a his3∆1 [leu2∆0] ura3∆0 met15∆0 SPT5 DamP rrp6∆::CgLEU2* | PCR amplification from pBS1763 using oligonucleotides LD1222 and LD1223 | YDL2537 | This work |
| YDL2577 | *SPT5 DAmP spt4∆ rrp6∆* | *Mat a [his3∆1] [leu2∆0] ura3∆0 met15∆0 SPT5 DAmP spt4∆::CgHIS3 rrp6∆::CgLEU2* | PCR amplification from pBS1763 using oligonucleotides LD1222 and LD1223 | YDL2574 | This work |
| YDL2576 | *ngl2∆* | *Mat a his3∆1 leu2∆0 lys2∆0 ura3∆0 ngl2∆::KanMX* |  |  | Euroscarf |
| YDL2540 | *SPT5::TAP* | *Mat a his3∆1 leu2∆0 met15∆0 ura3∆0 SPT5::TAP-HIS3MX6* |  |  | [3] - Open Biosystems |
| YDL2579 | *NRD1::HA SPT5::TAP* | *Mat a [his3∆1] leu2∆0 met15∆0 [ura3∆0] NRD1::HA SPT5::TAP-HIS3MX6* | PCR amplification from genomic DNA of YDL2540 using oligonucleotides LD2017 and LD2018 | YDL1678 | This work |
| YDL2594 alias YJL046 | *SPT5::HA* | *leu ura SPT5::HA-HIS3* |  |  | [5] |
| YDL2595 alias YJL047 | *SPT5∆CTR(1-15)::HA* | *leu ura SPT5∆CTR::HA-HIS3* |  |  | [5] |
| YDL2599 | *SPT5::HA NRD1::TAP* | *leu [ura] SPT5::HA-HIS3 NRD1::TAP-URA3* | PCR amplification from pBS1539 using LD2090 and LD2091 | YDL2594 alias YJL046 | This work |
| YDL2601 | *SPT5∆CTR::HA NRD1::TAP* | *leu ura SPT5∆CTR::HA-HIS3* | PCR amplification from pBS1539 using LD2090 and LD2091 | YDL2595 alias YJL047 | This work |
| YDL2604 | *RPA190::TAP* | *Mat a his3∆1 leu2∆0 [ura3∆0] met15∆0 RPA190::TAP-URA3* | PCR amplification from pBS1539 using LD2093 and LD2094 | BY4741 | This work |
| YDL2606 | *SPT5 ::HA RPA190 ::TAP* | *leu [ura] SPT5::HA-HIS3 RPA190::TAP-URA3* | PCR amplification from pBS1539 using LD2093 and LD2094 | YDL2594 | This work |
| YDL2608 | *SPT5∆CTR::HA RPA190::TAP* | *leu [ura] SPT5ΔCTR::HA-HIS3 RPA190::TAP-URA3* | PCR amplification from pBS1539 using LD2093 and LD2094 | YDL2595 | This work |
| YDL2536 | *SPT4::TAP* | *Mat a his3∆1 leu2∆0 met15∆0 ura3∆0 SPT4::TAP-HIS3MX6* |  |  | [3] - Open Biosystems |
| YDL2568 | *SPT5::TAP spt4∆* | *Mat a his3∆1 [leu2∆0] met15∆0 ura3∆0 SPT5::TAP-HIS3MX6 spt4∆::CgLEU2* | PCR amplification from pBS1763 plasmid using oligos LD1961 and LD1962 | YDL2540 | This work |
| YDL1076 | *RPA190::TAP* | *Mat a his3∆1 leu2∆0 met15∆0 ura3∆0 RPA190::TAP-HIS3MX6* |  |  | [3] - Open Biosystems |

Oligonucleotides used for the construction of yeast strains

| **Primer** | **Sequence (5’-3’)** |
| --- | --- |
| LD1222 | ATAGACGAAATAGGAACAACAAACAGCTTATAAGCACCCAATAAGTGCGTTCACAGGAAACAGCTATGACC |
| LD1223 | GGGGGAGCCATAACTCCATGACACAGATATTCGATTAGATGAATTTAGAGGGTTGTAAAACGACGGCCAGT |
| LD1961 | CACCTGGCCACATTCAGTTTGGCAAAAGCGAACGAGGTACAGTGTAAGAGCACAGGAAACAGCTATGACC |
| LD1962 | AGTAAAAAAAATTCATTACTATTATACATGTGATATCAGAACGGAAGGTTGTTGTAAAACGACGGCCAGT |
| LD2017 | CGGCCAAGGTACTGGTGC |
| LD2018 | ACGTATTGTCTACAGCGGCAAG |
| LD2090 | GAATATGCTTAACCAACAGCAGCAGCAACAACAACAAAGCTCCATGGAAAAGAGAAG |
| LD2091 | TTTTATGTACTATGAGCAAATAAAGGGTGGAGTAAAGATCTACGACTCACTATAGGG |
| LD2093 | GGGTTCATTTGATGTGTTAGCAAAGGTTCCAAATGCGGCTTCCATGGAAAAGAGAAG |
| LD2094 | AATATTAAATCGTAATAATTATGGGACCTTTTGCCTGCTTTACGACTCACTATAGGG |

**Oligonucleotides used for qPCR amplicons in ChIP analysis**

| **Amplicon** | **5’ primer** | **Sequence (5’-3’)** | **3’ primer** | **Sequence (5’-3’)** |
| --- | --- | --- | --- | --- |
| 1 | LD638 | TCGCGTATGGTCACCCACTAC | LD639 | GTTGCGGCCATATCTACCAGA |
| 3 | LD607 | GCAGTATTGAGACCATGAGAGTAGCA | LD608 | TCCAAATGTAAAATGGCCTATCG |
| 6 | LD609 | AAAGCAGTTGAAGACAAGTTCGAA | LD610 | GACTCTCTCCACCGTTTGACG |
| 5’-ETS | LD613 | GCGAAGGATTTGGTGGATTA | LD614 | TCACGGAATGGTACGTTTGA |
| 7 | LD611 | CTTGTCTCAAAGATTAAGCCATGC | LD612 | ACCACAGTTATACCATGTAGTAAAGGAACT |
| 9 | LD644 | GGTCTGTGATGCCCTTAGACG | LD645 | AGTTTCACAAGATTACCAAGACCTCTC |
| 10 | LD646 | AATATTAAAAACTTTCAACAACGGATCTCT | LD647 | CGATGATTCACGGAATTCTGC |
| 11 | LD648 | GGTGGTAAATTCCATCTAAAGCTAAATATT | LD649 | CACGTACTTTTTCACTCTCTTTTCAAA |
| 13 | LD652 | GGAGGAGTTATCTTTTCTTCTTAACAGCT | LD653 | AAGGTGCTGGCCTCTTCCA |
| 14 | LD654 | AAAGAAGACCCTGTTGAGCTTGA | LD655 | GTATTTCACTGGCGCCGAA |
| 17 | LD658 | TACGATGAGGATGATAGTGTGTAAGAGTG | LD659 | CTCTTTCAACCCATCTTTGC |
| 18 | LD660 | CTCATTTCCTATAGTTAACAGGACATGC | LD661 | TTCACTTGTCTCTTACATCTTTCTTGG |
| snR33 | LD1631 | CGGAACGGTACATAAGAATAGAAGAG | LD1632 | TTCAATCTCTGCTCCTCCAAAC |

All oligonucleotides were previously used in [6] except LD1631 and LD1632, which are described in [7].

Oligonucleotides used for Northern Blot analysis

| **Primer** | **Sequence (5’-3’)** |
| --- | --- |
| LD339 | GGCCAGCAATTTCAAGTTA |
| LD359 | TTGTTACCTCTGGGCCC |
| LD471 | CGGTTTTAATTGTCCTA |
| LD871 | CATGGCTTAATCTTTGAGAC |
| LD906 | TGAGAAGGAAATGACGCT |
| LD1099 | CTCCGCTTATTGATATGC |
| LD1142 (PGK1) | ACCGTTTGGTCTACCCAAGTGACAAGCCAAGACA |
| LD1290 (SCR1) | ATCCCGGCCGCCTCCATCAC |
| LD1299 (GRE1-R) | CGCACATCCATGTGTCAAGA |
| LD1300 (GRE1-F) | TAATACGACTCACTATAGGGCTGACAGTTCCGGTAAGTTCTT |

Oligonucleotides used for RTqPCR analysis

| **Primer** | **Amplicon name** | **Sequence (5’-3’)** |
| --- | --- | --- |
| LD2021 | SPT5 | TACAGATGATGCCCAAGCAA |
| LD2022 | SPT5 | CGTCCTCATCATCATCATCG |
| LD714 | ACTIN 1 | CGTTCCAATTTACGCTGGTT |
| LD715 | ACTIN 1 | AGCGGTTTGCATTTCTTGTT |

**SUPPLEMENTAL MATERIALS AND METHODS REFERENCES**

1. Conrad NK, Wilson SM, Steinmetz EJ, Patturajan M, Brow DA, et al. (2000) A yeast heterogeneous nuclear ribonucleoprotein complex associated with RNA polymerase II. Genetics 154: 557-571.

2. Steinmetz EJ, Conrad NK, Brow DA, Corden JL (2001) RNA-binding protein Nrd1 directs poly(A)-independent 3'-end formation of RNA polymerase II transcripts. Nature 413: 327-331.

3. Ghaemmaghami S, Huh WK, Bower K, Howson RW, Belle A, et al. (2003) Global analysis of protein expression in yeast. Nature 425: 737-741.

4. Breslow DK, Cameron DM, Collins SR, Schuldiner M, Stewart-Ornstein J, et al. (2008) A comprehensive strategy enabling high-resolution functional analysis of the yeast genome. Nat Methods 5: 711-718.

5. Zhou K, Kuo WH, Fillingham J, Greenblatt JF (2009) Control of transcriptional elongation and cotranscriptional histone modification by the yeast BUR kinase substrate Spt5. Proc Natl Acad Sci U S A 106: 6956-6961.

6. Wery M, Ruidant S, Schillewaert S, Lepore N, Lafontaine DL (2009) The nuclear poly(A) polymerase and Exosome cofactor Trf5 is recruited cotranscriptionally to nucleolar surveillance. The RNA J 15: 406-419.

7. Kim M, Vasiljeva L, Rando OJ, Zhelkovsky A, Moore C, et al. (2006) Distinct pathways for snoRNA and mRNA termination. Mol Cell 24: 723-734.
